# The effect of isoniazid preventive therapy on incidence of tuberculosis among HIV-infected clients under pre-ART care, Jimma, Ethiopia: a retrospective cohort study

**DOI:** 10.1186/s12889-015-1719-0

**Published:** 2015-04-10

**Authors:** Lelisa Fekadu Assebe, Hailemariam Lemma Reda, Alem Desta Wubeneh, Wondwossen Terefe Lerebo, Saba Maria Lambert

**Affiliations:** Ethiopian Federal Ministry of Health, Disease Prevention and Control Directorate, Addis Ababa, Ethiopia; Department of Public Health, College of Health Sciences, Mekelle University, Mekelle, Ethiopia; Dermatology Department, All African Leprosy Tuberculosis and Rehabilitation Training Hospital, Addis Ababa, Ethiopia

**Keywords:** Isoniazid preventive therapy, Incidence, TB, IPT Effect, Pre-ART care

## Abstract

**Background:**

Tuberculosis (TB) is a major public health problem that accounts for almost half a million human immunodeficiency virus (HIV) associated deaths. Provision of isoniazid preventive therapy (IPT) is one of the public health interventions for the prevention of TB in HIV infected individuals. However, in Ethiopia, the coverage and implementation of IPT is limited. The objective of this study is to compare the incidence rate of TB, TB-free survival time and identify factors associated with development TB among HIV-infected individuals on pre-ART follow up.

**Methods:**

A retrospective cohort study was conducted from January, 2008 to February 31, 2012 in Jimma hospital. Kaplan-Meier survival plots were used to calculate the crude effect in both groups on TB-free survival probabilities and compared using the log rank test. A Cox proportional hazard model was used to identify predictors of TB.

**Result:**

A total of 588 patients on pre-ART care (294 IPT and 294 non-IPT group) were followed retrospectively for a median duration of 24.1 months. The median CD_4_^+^ cell count was 422 cells/μl (IQR 344 – 589). During the follow up period, 49 individuals were diagnosed with tuberculosis, giving an overall incidence of 3.78 cases per 100 person year (PY). The incidence rate of TB was 5.06 per 100 PY in non-IPT group and 2.22 per 100 PY in IPT user group. Predictors of higher TB risk were: being on clinical WHO stage III/IV (adjusted hazard ratio (AHR = 3.05, 95% confidence interval (CI): 1.61, 5.81); non-IPT user (AHR = 2.02, 95% CI: 1.04, 3.92); having CD_4_^+^ cell count less than 350 cells/μl (AHR = 3.16, 95% CI: 1.04, 3.92) and between 350–499 cells/μl, (AHR = 2.87; 95% CI: 1.37 - 6.03) and having episode of opportunistic infection (OI) in the past (AHR = 2.41, 95% CI: 1.33-4.34).

**Conclusion:**

IPT use was associated with fifty percent reduction in new cases of tuberculosis and probability of developing TB was higher in non-IPT group. Implementing the widespread use of IPT has the potential to reduce TB rates substantially among HIV-infected individuals in addition to other tuberculosis prevention and control effort in resource limited settings.

## Background

Tuberculosis remains a major public health problem throughout the world. Globally almost one third of the world population is estimated to be latently infected with *Mycobacterium tuberculosis* and hence at risk of developing active TB disease [1].

The TB epidemic has been fuelled by HIV co-infection, one increasing the other’s impact.

HIV infection, through waning of the immune system, increases the susceptibility mycobacterium tuberculosis infection and progression to active disease [2]. The TB epidemic is further, aggravated by drug resistance, social inequalities, limited TB control efforts and limited access to health care services [3,4]. The risk of developing TB is between 20 to 37 times greater in HIV infected individuals when compared to immunocompetent individuals [5]. As a result, TB is the commonest infection and common cause of death among HIV-infected individuals [6].

According to the world health organization (WHO) 2013 global TB report, there were an estimated 8.6 million new TB cases and 1.3 million TB related deaths (a quarter of these deaths were associated with HIV). More than half of these estimated TB cases occurred in Asia and western pacific regions. HIV positive TB cases accounts for 1.1 million (13% of all TB cases) among patients with documented HIV test result. Majority of the co-infected cases (75%) were in Africa. Nearly 57% of HIV positive TB cases were on ART and 520,000 HIV-positive people were reported to have received TB preventive therapy. South Africa accounts for the highest proportion of IPT coverage (71%) [6]. This report also shows that Ethiopia ranks seventh amongst the world’s 22 high TB burden countries in the world and TB is the second leading cause of hospital death in the country [6,7]. In Ethiopia, 79% of HIV infected individuals were screened for active TB, of whom 15% had TB/HIV co-infection. Only 19% of the HIV positive clients without active TB were provided with IPT in 2010 [7,8]. This dual epidemic of HIV/AIDS and TB is a growing concern that challenges the Ethiopian government’s efforts towards prevention and control of both disease [7,9,10].

To reduce the burden of TB among HIV infected individuals, the country had adopted twelve key TB/HIV collaborative activities recommended by World Health Organization (WHO) [5-7,11]. Provision of ART significantly reduces TB/HIV related mortality and risk of incident TB [5,12]. However ART alone isn’t adequate enough in reducing tuberculosis risk in HIV infected individuals, hence implementation of other TB specific intervention to further reduce the risk of TB in HIV-infected individuals is required [5,12-14].

Multiple studies have demonstrated the effectiveness of IPT in adults [9,11,13,15-18]. Meta-analyses of randomized controlled trials have shown that IPT reduces the risk of TB by 33% overall and by 64% when targeted to HIV infected individuals who had a positive tuberculin skin test [13]. An earlier (1998) systematic review of four placebo controlled randomized trials, concluded that approximately one half of active tuberculosis cases were reduced with use of IPT [19]. Cohort and case control studies have shown that IPT provision for HIV-infected adults significantly reduce tuberculosis incidence independently or with coadministration of highly active antiretroviral therapy (HAART) [11,15-17,20,21]. In contrary three cohort studies conducted in Brazil, Tanzania, and Kenya showed IPT had no significant effect on the reduction of TB among HIV-infected individuals [22-24].

In spite of good evidence of IPT uses and the global recommendation as part of standard care for all HIV infected individuals after ruling out active TB, it was noted that the coverage and implementation had been slow in many countries including Ethiopia [7,9,18]. These problems were linked with concern about development of isoniazid related resistance, drug side effects, providers’ attitude on its effectiveness, failure to consistently use TB screening algorithms and shortage of drug supplies [18,25,26]. These factors may affect both the perception and attitude of the policy makers, health care providers and clients towards TB preventive therapy. Studies that have shown inconsistent results of IPT protection against TB may cause further misunderstanding. A critical review of barriers related to the health system and client related issues should be carried out in order to develop nation specific strategies that are essential to foster the implementation of IPT program. In Ethiopia the effect of IPT on the incidence of TB and the determinants associated with TB disease among HIV infected individuals (pre-ART) are not well studied. Furthermore little is known on TB free survival probability among IPT user and non-users. Therefore this study is aimed at investigating IPT effect and identifying determinants factors for TB among HIV-infected individual in Jimma University Specialized Hospital (JUSH), in southwest Ethiopia. The study was based on the hypothesis that there is no significant difference in TB incidence between IPT user and non-user among HIV-infected individuals.

## Methods

### Study setting and design

Jimma University Specialized Hospital (JUSH) is a government hospital, rendering its referral and specialized medical services to more than 15 million populations in south western Ethiopia. The hospital has enrolled more than 7,400 patients to HIV care and follow-up, among these 3,476 were on pre-ART care follow-up during the study period. A retrospective cohort study design was employed in this study.

### Study period and population

The source population was HIV infected individuals enrolled in pre-ART care follow-up, whose age was between 15 and 64 years. The study population was a sample of the source population who was under care during the period January 2008 to February 2012. Exclusion criteria were incomplete patient records, past history of TB, active TB, or on treatment for TB.

### Sample size determination

The sample size was determined using sample size determination formula for independent cohort studies using Epi-info version 7, for IPT and non-IPT group. Incidence of TB among IPT group 2.2% and among non-IPT group 7.5% were taken from previous study [27]. Taking ratio of exposed to unexposed 1:1 with 80% power and 5% type I error, the resulting total sample size was 588.

The total number of adult patients in pre-ART care was obtained from the pre-ART register (N = 3476) and patients were categorized according to IPT (n_1_ = 420) and non-IPT group (n_2_ = 2648). Sampling frame for IPT and non-IPT group was prepared by assigning sequentially unique number to each patient medical record in each groups separately; simple random sampling method was employed to select participant records from both groups using computer generated random numbers.

### Variables and source of data

The main outcome variable was tuberculosis. Diagnosis of TB in HIV positive individuals in this analysis was based on the national guidelines for clinical and programmatic management of TB, TB/HIV and leprosy in Ethiopia. Smear-positive pulmonary TB was established if at least one sputum smear examination positive for Acid-fast bacilli (AFB). The algorithm for smear negative pulmonary TB diagnosis required at least three sputum specimens negative for AFB, radiologic abnormalities consistent with active tuberculosis, decision by a clinician to treat with full course of Anti-TB chemotherapy, or patient with AFB smear-negative sputum which is culture-positive for MTB. Extra pulmonary TB diagnosis was based on histological or strong clinical evidence consistent with active extra pulmonary, with the clinician’s decision to treat the patient with a full course of anti-tuberculosis treatment or one specimen from an extra pulmonary site culture or smear positive for AFB. For this analysis, both pulmonary TB and extra pulmonary TB were included [7].

The primary exposures of interest were initiation of IPT. Prior to initiating IPT in HIV-infected individuals, it is important to screen for TB. As a screening tool, the Ethiopian guidelines suggest a four symptoms based clinical algorithm that comprises of current cough, night sweats, weight loss and fever. HIV infected individuals without any of the symptoms are unlikely to have active TB and should be offered IPT. IPT is provided for a period of six months, with monthly follow-up until the treatment course is completed [7]. However tuberculin skin test (TST) isn’t a requirement for IPT initiation in the country [7]. We extracted data on these and other predictor variables from standard national pre-ART register, follow up forms and other clinical records. These register and forms are regularly filled as part of a routine paper-based patient record system. Important data related to socio-demographic characteristics (age, sex, marital status, level of education, religion, residence, employment status). clinical, laboratory and IPT information (past OI, clinical WHO stage, CD_4_^+^ cells count, weight, other OI treatment initiation, Hgb, IPT and functional status, incident TB). Social condition and substance use (number of people in the household, tobacco use, alcohol use, soft drugs and hard drugs) were extracted by trained nurses.

### Data processing and analysis

Completed questionnaires were coded, entered and analyzed using STATA version 11.1 software. Each variable was checked for missing and coding problem. Descriptive statistics was used to summarize the characteristics of the cohort.

TB incidence rate was calculated for IPT and non-IPT group in HIV-infected individuals. The TB free survival probability (survival analysis) was calculated in months using the time interval between dates registered on pre-ART care or IPT prophylaxis initiation to date of TB diagnosis or censoring. Subject was censored on the date of completion of the study, died, lost to follow-up, transferred out and ART initiated; whichever occurred first. For subjects lost to follow-up, transferred out and died, the date of their last follow-up visit was used as the censoring date.

Kaplan-Meier survival plots were used to calculate the crude effect of both groups on TB-free survival probabilities and compared using the log rank test. Crude hazard ratio test was used for inclusion of variables into multivariate analysis with cut off p-value ≤ 0.25. Cox proportional hazard model was used to identify factors associated with incidence of TB and a p-value ≤ 0.05 declares the significance of the variables at 95% confidence level.

The multivariate model was built using purposeful selection with backward elimination method [28]. Confounding was checked and percentage change in the regression coefficients (β) less than 20% reveals absence of confounder [28]. Interaction for the main effect model was checked and Partial likelihood ratio test has p-value > 0.05 and VIF < 10 indicating non-existence of multicollinearity among the variables in this study. A statistical and graphical test was used to assess the proportional hazard assumptions and the result showed that none of the predictors violated the proportional hazard assumptions and there was no strong evidence of non-fit.

### Ethical consideration

The research was approved by Institutional Review Board of Mekelle University, College of Health Science (MU-CHS) with reference number ERC 0228/2013. A support letter referred with BEFO/HBTFH/1-8/2234 was sought from Oromia Regional Health Bureau. Jimma University Specialized Hospital was informed about the objective of the study and written permission was obtained from the hospital administrator before starting data collection.

All the collected patient information was stored anonymous and data was kept confidential. More over personal identifiers was not included in the data collection forms.

## Results

A total of 588 HIV infected individuals (294 IPT and 294 non- IPT cohort) were followed for a median duration of 22.61 (IQR 17.13-28.56) months in the IPT and 26.68 (IQR 14.66-43.66) months in the non-IPT group (Table 1). Among the IPT group, majority of the patients 180 (61.43%) were female. Similarly among the non-IPT group, females accounted for 62.59%. At the time of enrollment to pre-ART care, there were 124 (42.18%) and 133 (45.24%) patients under age of 29 years in the IPT and non-IPT groups, respectively (Table 1).

**Table 1:** **Demographic, socio-economic, substance use characteristics of the study participants in pre-ART follow up, JUSH, 2013, N = 588**

**Variables**	**Category**	**pre-ART**		**Total**
		**IPT group no (%)**	**Non-IPT group no (%)**	
**Age group (years)**	15-29	124 (42.18%)	133 (45.24%)	257 (43.71%)
	30-39	108 (36.73%)	93 (31.63%)	201 (34.18%)
	39+	62 (21.09%)	68 (23.13%)	130 (22.11%)
**Sex (n = 587)**	Female	180 (61.43%)	184 (62.59%)	364 (62.01%)
	Male	113 (38.57%)	110 (37.41%)	223 (37.99%)
**Residence (n = 585)**	Urban	201 (69.07%)	201 (68.37%)	402 (68.72%)
	Rural	90 (30.93%)	93 (31.63%)	183 (31.28%)
**Marital status**	Never Married	51 (17.35%)	54 (18.37%)	105 (17.86%)
	Married	159 (54.08%)	156 (53.06%)	315 (53.57%)
	Separated	31 (10.54%)	21 (7.14%)	52 (8.84%)
	Divorced	31 (10.54%)	29 (9.86%)	60 (10.20%)
	Widowed	22 (7.48%)	34 (11.56%)	56 (9.52%)
**Level of education (n = 585)**	No education	53 (18.21%)	86 (29.25%)	139 (23.76%)
	Primary	123 (42.27%)	111 (37.76%)	234 (40.00%)
	Secondary	86 (29.55%)	71 (24.15%)	157 (26.84%)
	Tertiary	29 (9.97%)	26 (8.84%)	55 (9.40%)
**Religion (n = 587)**	Protestant	35 (11.95%)	50 (17.01%)	85 (14.48%)
	Orthodox	174 (59.39%)	133 (45.24%)	307 (52.30%)
	Muslim	78 (26.62%)	104 (35.37%)	182 (31.01%)
	Catholic	6 (2.05%)	7 (2.38%)	13 (2.21%)
**Employment status (n = 585)**	Employed	116 (39.86%)	104 (35.37%)	220 (37.61%)
	Unemployed	175 (60.14%)	190 (64.63%)	365 (62.39%)
**People/household (n = 583)**	<=2	89 (30.38%)	92 (31.72%)	181 (31.05%)
	>2	204 (69.62%)	198 (68.28%)	402 (68.95%)
**Substance use**				
**Tobacco**	Yes	37 (12.63%)	57 (19.39%)	94 (16.01%)
	No	256 (87.37%)	237 (80.61%)	493 (83.99%)
**Alcohol**	Yes	64 (21.77%)	74 (25.17%)	138 (23.47%)
	No	230 (78.23%)	220 (74.83%)	450 (76.53%)
**Soft drugs** ^**1**^	Yes	92 (31.29%)	110 (37.41%)	202 (34.35%)
	No	202 (68.71%)	184 (62.59%)	386 (65.65%)
**Hard drugs** ^**2**^	Yes	31 (10.58%)	33 (11.26%)	64 (10.92%)
	No	262 (89.42%)	260 (88.74%)	522 (89.08%)
**Follow up time median (IQR)**	Over all	24.08 (IQR = 15.63-36.98) month		
	IPT	22.61 (IQR = 17.13-28.56) month		
	Non-IPT	26.68 (IQR = 14.66-43.66) month		

^1^khat, “shisha”, pills ^2^cocaine, morphine, intravenous drug use.

### Clinical and laboratory characteristics, and follow-up outcome

Nearly half of the patients had at least one episode of opportunistic illness in the past, 145 (49.49%) of them were from IPT and 126 (42.86%) were from non-IPT group. Out of the total study participants, majority of IPT group patients were in clinical WHO stage I/II 256 (87.07%). The median CD4^+^ cell count was 422 cells/μl (IQR 344 – 589) for the entire cohort, 415 cells/μl (IQR 328–786) for IPT group, 444 cells/μl (IQR 357–613) for non-IPT group.

Out of 237 (40.51%) patients who had CD4^+^ cell count between 350–499 cells/μl, 121 (41.44%) were in IPT group and 116 (39.59%) of them were in non-IPT group (Table 2).

**Table 2:** **Clinical and laboratory characteristics, and follow-up outcome of the study participants on Pre-ART follow up, JUSH, 2013, N = 588**

**Variables**	**Category**	**Pre-ART**		**Total**
		**IPT group No (%)**	**Non-IPT group No (%)**	
**Past opportunistic infection(n = 587)**	Yes	145 (49.49%)	126 (42.86%)	271 (46.17%)
	No	148 (50.51%)	168 (57.14%)	316 (53.83%)
**WHO clinical stage**	Stage I/ II	256 (87.07%)	227 (77.21%)	483 (82.14%)
	Stage III/ IV	38 (12.93%)	67 (22.79%)	105 (17.86%)
**Functional status**	Working^1^	270 (91.84%)	250 (85.03%)	520 (88.44%)
	Ambulatory^2^	22 (7.48%)	43 (14.63%)	65 (11.05%)
	Bedridden^3^	2 (0.68%)	1 (0.34%)	3 (0.51%)
**OI treatment other than INH (n = 584)**	Given^4^	143 (49.14%)	124 (42.32%)	267 (45.72%)
	Not given	148 (50.86%)	169 (57.68%)	317 (54.28%)
**Hemoglobin level (n = 532)**	<=10 mg/dl	16 (5.90%)	19 (7.28%)	35 (6.58%)
	>10 mg/dl	255 (94.10%)	242 (92.72%)	497 (93.42%)
**CD**_**4**_ **cells count (cells/μl) (n = 585)**	<350	84 (28.77%)	69 (23.55%)	153 (26.15%)
	350 – 499	121 (41.44%)	116 (39.59%)	237 (40.51%)
	> = 500	87 (29.79%)	108 (36.86%)	195 (33.33%)
**Weight (kg)**	<40	3 (1.02%)	13 (4.42%)	16 (2.72%)
	40-49	81 (27.55%)	87 (29.59%)	168 (28.57%)
	50-60	117 (39.63%)	129 (43.88%)	246 (41.84%)
	>60	93 (31.63%)	65 (22.11%)	158 (26.87%)
**Follow-up outcome**	Alive	181 (61.56%)	105 (35.71%)	286 (48.64%)
	Lost to follow up	66 (22.45%)	109 (37.07%)	175 (29.76%)
	Entry to ART	20 (6.8%)	19 (6.46%)	39 (6.63%)
	TB	13 (4.42%)	36 (12.24%)	49 (8.83%)
	Other^5^	14 (4.76%)	25 (8.50%)	39 (6.63%)
**Median CD**_**4**_ **cells count (cells/μl)**		415 (IQR 328–786)	444 (IQR 357–613)	422 (IQR 344–589)

^1^Working: able to perform usual work in and out of the house.

^2^Ambulatory: able to perform activities for daily living.

^3^Bedridden: unable to perform activities for daily living.

^4^Cotrimoxazole, Fluconazole.

^5^Transferred out and dead.

### Comparison of TB incidence rate and TB-free survival time

There were 49 incident TB cases in this study cohort during 1297.5 PY of observation and the overall tuberculosis incidence rate was 3.78 per 100 PY of follow up (95% CI: 2.85, 4.99 cases per 100 PY). The TB incidence rate in the IPT group was 2.22 new cases of TB per 100 person-year (95% CI: 1.29, 3.82 cases per 100 PY) and 5.06 per 100 person-year (95% CI: 3.65, 7.02 cases per 100 PY) for non IPT group (Table 3). Individuals taking isoniazid preventive therapy were at lower risk of developing tuberculosis as compared to their counter parts with adjusted hazard ratio (AHR) of 2.02 (95% CI: 1.04 -3.92) (Table 4).

**Table 3:** **Incidence of tuberculosis per 100 person-year according to exposure category among HIV infected individuals on pre-ART follow up in, JUSH, 2013, N = 588**

**Group**	**No. of cases of tuberculosis**	**No. of PY**	**Incidence rate of TB per 100 PY (95% CI per 100 PY)**
IPT	13	586.16	2.22 (1.29- 3.82)
non-IPT	36	711.41	5.06 (3.65 - 7.02)
**Overall TB incidence**	49	1297.50	3.8 (2.85 - 4.99)

**Table 4:** **Bivariate and multivariate cox proportional hazards model of incident TB among HIV infected individuals on pre-ART follow-up in, JUSH, 2013, N = 584**

**Variables**	**Category**	**Crude hazard ratio (95% CI)**	^**¥**^ **Adjusted hazard ratio (95% CI)**
**Age group (years)**	15-29	1.00	
	30-39	0.88 (0.48 1.62)	
	39+	0.38 (0.15 0.98)*	
**Sex (N = 587)**	Female	1.00	
	Male	0.61 (0.35 1.06)	
**Marital status**	Never married	1.00	
	Married	0.56 (0.28 1.12)	
	Separated	0.83 (0.29 2.35)	
	Divorced	0.72 (0.27 1.91)	
	Widowed	0.23 (0.05 1.02)	
**Level of education (N = 585)**	Primary	1.00	
	No education	1.85 (0.95 3.63)	
	Secondary	1.38 (0.67 2.85)	
	Tertiary	0.63 (0.15 2.74)	
**People per household (N = 583)**	<=2	1.00	
	>2	1.54 (0.76 3.09)	
**Tobacco use**	Yes	1.00	
	No	0.66 (0.35 1.25)	
**Alcohol use**	Yes	1.00	
	No	0.68 (0.37 1.23)	
**Past opportunistic infection (N = 587)**	No	1.00	1.00
	Yes	2.57 (1.43 4.61)**	2.41 (1.33 4.34)
**WHO clinical stage**	Stage I/ II	1.00	1.00
	Stage III/ IV	3.39 (1.90 6.02)**	3.05 (1.61 5.81)**
**Functional status**	Working	1.00	
	Ambulatory	1.29 (0.58 2.88)	
	Bedridden	5.65 (0.77 41.32)	
**IPT prophylaxis®**	Yes	1.00	1.00
	No	1.95 (1.02 3.74)	2.02 (1.04 3.92)*
**CD**_**4**_^**+**^ **cells count (cells/μl) (N = 585)**	> = 500	1.00	1.00
	350 – 499	3.06 (1.47 6.37)**	2.87 (1.37 6.03)**
	<350	2.34 (1.02 5.39)*	3.16 (1.04 3.92)**

*Statistically significant at p ≤ 0.05 **statistically significant at p ≤ 0.01.

®Exchanging the reference at adjusted level of analysis results in (aHR = 0.5).

^¥^Global test of proportional-hazards assumption for predictors fitted to Cox proportional hazard model was not significant (df = 5, ch^2^ = 7.04, p = 0.218).

**Figure 1:**
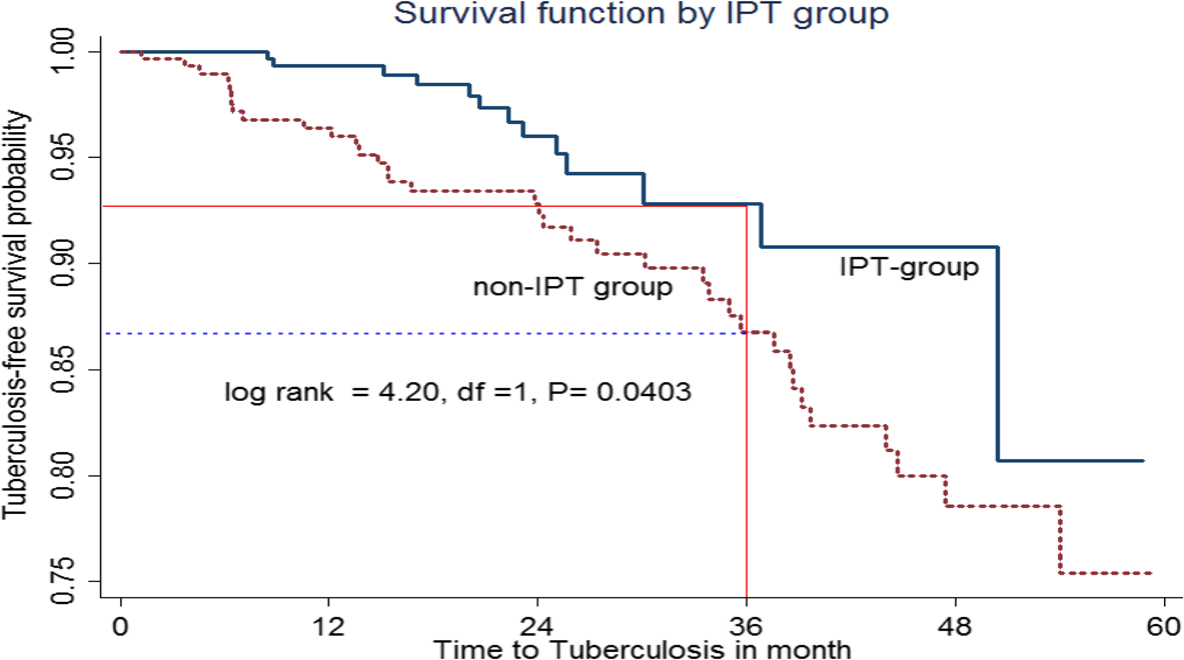
**Kaplan-Meier estimate of tuberculosis-free survival probability in IPT and non-IPT group, JUSH, 2013.**

The TB free survival proportion for the entire cohort over the follow up period was 0.77 and the TB-free survival probability in the IPT user group was significantly higher than the non-IPT user group throughout the study (log rank statistic = 4.20, df =1, P = 0.0403) (Figure 1). Furthermore Figure 1 shows the cumulative TB-free survival at thirty six months of follow-up was approximately 93% in IPT and 87% non-IPT user groups.

### Predictors of tuberculosis incidence

HIV infected individuals who didn’t received IPT during the study period had two times higher risk of developing TB as compared to those who received IPT, after adjusting for past opportunistic illness, clinical WHO stage and CD_4_^+^ cell count (AHR = 2.02; 95% CI: 1.04-3.92). The presence of past episode of opportunistic illness were also associated with increased hazard of developing TB after controlling for IPT use, clinical WHO stage and CD_4_^+^ cell count (AHR = 2.41; 95% CI: 1.33-4.34) (Table 4).

Individuals with clinical WHO stage III/IV had nearly 3.1 times increased risk of developing TB as compared to patients in clinical WHO stage I/II after controlling for past opportunistic illness, IPT use and CD_4_^+^ cell count (AHR = 3.05; 95% CI: 1.61- 5.81). Compared to those presenting with CD_4_^+^ cell count > =500 cells/μl, patients with CD_4_^+^ cell count less than 350 cells/μl had 3.2 times (AHR = 3.16; 95% CI: 1.04 - 3.92) and between 350 – 499 cells/μl had 2.9 times (AHR = 2.87; 95% CI: 1.37 - 6.03) higher hazard of developing TB after controlling for past opportunistic illness, clinical WHO stage and IPT use (Table 4).

In summary, HIV infected individuals with advanced clinical WHO stage, non-IPT use, low CD4+ cell count and history of past episode of opportunistic illness, were more likely to have higher incidence of TB (Table 4).

## Discussion

The overall incidence of tuberculosis was 3.78 per 100PY in the entire follow-up period. This finding was consistent with a cohort study in Zambia that shows 3.6 per 100 PY [29]. However, this result was higher than study done in Brazil and Ethiopia with TB incidence 2.28 per 100 PY 2.6 per 100 PY, respectively [17,22]. This inconsistency might be due to smaller number of individuals receive IPT, some of the study participants were taking ART and difference in source population, as the source population of those study includes all age groups in the latter studies [17,22].

In our study the incidence of TB was higher among patients with no prophylaxis as compared to patients with IPT prophylaxis. This finding was in agreement with studies done in Namibia, Brazil and Ethiopia [9,11,16,17,21]. However, a cohort study in Thailand and clinical trial in Kenya showed, there was no difference in TB incidence whether a patient took IPT or not [24,30]. This difference might be explained by limited scope focusing only to see incidence of pulmonary type of tuberculosis cases in Thailand study [30]. Similarly, the benefit of isoniazid might be limited in the Kenya study due to high rate of transmission of new infection and insufficient duration of isoniazid provision for individuals with relatively advanced immune-suppression and non-adherence to therapy [24]. Our results suggest that the difference in incidence of tuberculosis might be attributed to the use of IPT that prevents progression of latent infection to active disease.

This study showed that IPT offers a long term benefit in HIV-infected individuals against tuberculosis with a higher TB free survival particularly during the first three years of follow up among IPT users. Therefore the use of IPT during pre-ART care shows higher decrease in cumulative risk of TB disease in HIV-infected patients. This finding agrees with observational study conducted in Spain [31].

Similarly, TB-free survival time was higher in IPT group than non-IPT group. This finding was supported by studies done in South Africa and Namibia that showed higher TB-free survival probability among IPT-group [15,21]. In contrary, recent trial study conducted in Botswana shows that shorter median time to develop TB among individuals who took IPT for 6 month [32]. This variation might be due to higher risk of re-infection to tuberculosis and inadequate power in latter study.

Our study revealed IPT use has contributed to a higher reduction in hazard of acquiring tuberculosis than non-IPT use. This finding was consistent with pooled estimate of four trials carried out in Uganda, Haiti, USA and Kenya that showed approximately the same level of reduction of TB in adults infected with HIV [19]. It was also in agreement with other studies conducted in Namibia, Brazil and Ethiopia [16,17,21]. But a recent meta-analysis of ten trails revealed that IPT use was associated with a smaller reduction of TB among HIV infected individuals [33]. In contrary, other studies conducted in Brazil, Tanzania and Kenya showed that insignificant effect of IPT on the reduction of TB [22-24]. The smaller reduction of TB in the meta-analysis might be due to inclusion of studies from resource rich countries with low TB prevalence and study design difference [33]. The disagreement with Brazil, Tanzania and Kenya may be due to difference in number of individuals taking IPT; high rate of transmission of new infection and insufficient duration of the prophylaxis in subjects with relatively advanced immune-suppression were among the possible reason for absence of the IPT benefit in these studies [22-24].

This study showed that patients presenting with clinical WHO stages III and IV is associated with higher hazard of developing tuberculosis as compared to patients in clinical WHO stage I and II. The result was in agreement with study conducted in South Africa and Ethiopia [14,17,20]. This may be due to the fact that HIV weakens the immune system and lead, more opportunistic infections are likely to occur.

Our study also revealed that patients who suffered from opportunistic infection had higher hazard of developing TB as compared to those free of the infections in the past. Finding from a clinical trial in Uganda and case control study in Ethiopia agreed with our finding [11,34]. In this study, among the determinant factors, having lower CD_4_^+^ count were associated with increased relative hazard for developing TB. The risk of TB shows a higher increase when CD_4_^+^ cell counts fall below 350 cells/μl. This result is consistent with cohort studies that show gradual increase of the risk of TB when CD_4_^+^ cell count falls down [15,17,31].

### Strength and limitation of the study

This is one of the few studies in Ethiopia that explored association of IPT provision with the development of TB in individuals under pre-ART care. This study did have some limitations that are shared with most studies. The retrospective nature of the study design limited to include other factors that may influence the risk of TB (house-hold income, housing condition).

## Conclusion and recommendation

Provision of TB preventive therapy to all eligible HIV-infected individuals in high TB prevalence before they reach to severe form of HIV disease is an important approach to prevent and control tuberculosis. This study indicates that isoniazid preventive therapy confers a significant reduction in TB incidence among HIV infected individuals and adjustment for potential confounder didn’t alter the estimate of its effectiveness.

Efforts should be strengthened in implementing the widespread use of IPT among adult HIV-infected patients through integration with intensified case finding and development of operational guidelines for the implementation of IPT in HIV care and treatment settings.

HIV infected individuals with advanced immunosuppression and past episode of opportunistic infection were at higher risk of developing tuberculosis, which implies undertaking in depth TB screening and frequent follow up among these patient is critical in order to prevent and control tuberculosis. Further prospective studies might be needed to establish the optimal duration of its protective effect and the added benefit of isoniazid preventive therapy (IPT) among people receiving antiretroviral therapy should be established in the country.

## Abbreviations

AFB: Acid fast bacilli
AHR: Adjusted hazard ratio
CI: Confidence interval
HAART: Highly active anti-retroviral therapy
HIV: Human immunodeficiency virus
IPT: Isoniazid preventive therapy
IQR: Interquartile range
JUSH: Jimma university specialized hospital
MU-CHS: Mekelle university college of health science
OI: Opportunistic infection
PY: Person year
HR: Hazard ratio
TB: Tuberculosis
TST: Tuberculin skin test
UNAIDS: United nations programme on HIV/AIDS
WHO: World Health Organization
